# III–V nanowires on silicon (100) as plasmonic-photonic hybrid meta-absorber

**DOI:** 10.1038/s41598-021-93398-z

**Published:** 2021-07-05

**Authors:** Hyunseok Kim, Haneui Bae, Ting-Yuan Chang, Diana L. Huffaker

**Affiliations:** 1 Department of Mechanical Engineering, Massachusetts Institute of Technology, Cambridge, MA 02139 USA; 2 Department of Electrical and Computer Engineering, University of California Los Angeles, Los Angeles, CA 90095 USA; 3 Department of Molecular and Cellular Biology, Harvard University, Cambridge, MA 02138 USA; 4 Department of Electrical Engineering, University of Texas at Arlington, Arlington, TX 76019 USA

**Keywords:** Nanophotonics and plasmonics, Nanoscale devices, Nanoscale materials, Nanowires, Metamaterials, Nanowires, Silicon photonics

## Abstract

Integration of functional infrared photodetectors on silicon platforms has been gaining attention for diverse applications in the fields of imaging and sensing. Although III–V semiconductor is a promising candidate for infrared photodetectors on silicon, the difficulties in directly growing high-quality III–V on silicon and realizing functionalities have been a challenge. Here, we propose a design of III–V nanowires on silicon (100) substrates, which are self-assembled with gold plasmonic nanostructures, as a key building block for efficient and functional photodetectors on silicon. Partially gold-coated III–V nanowire arrays form a plasmonic-photonic hybrid metasurface, wherein the localized and propagating plasmonic resonances enable high absorption in III–V nanowires. Unlike conventional photodetectors, numerical calculations reveal that the proposed meta-absorber exhibits high sensitivity to the polarization, incident angle, wavelength of input light, as well as the surrounding environment. These features represent that the proposed meta-absorber design can be utilized not only for efficient infrared photodetectors on silicon but for various sensing applications with high sensitivity and functionality.

## Introduction

Infrared photodetectors on silicon platforms are extensively utilized in diverse applications, including optical communications, lidar, bio- and chemical sensing, and surveillance. Since silicon-based detectors exhibit cutoff wavelength at 1 µm, heterogeneously integrated photodetectors with absorber materials having smaller bandgap, such as SiGe and III–V compound semiconductors, are explored as promising candidates for extending the detection regime to longer wavelengths. While SiGe- or Ge-based photodetectors can cover only up to a wavelength of around 1.8 µm, III–V semiconductors can cover a much wider range, including short-wave infrared (SWIR), mid-wave infrared (MWIR), long-wave infrared (LWIR), and even terahertz regime by forming heterostructures, with the capability of high-speed operation due to high carrier mobility^1,2^. However, one of the biggest challenges for forming III–V photodetectors on silicon platforms is their integration difficulties. Due to the large mismatches in lattice constants and thermal expansion coefficients between III–V and silicon, direct growth of III–V materials on silicon induces high density of dislocations, degrading the detector performance by increased dark current and reduced quantum efficiency^3^. Therefore, heterointegration by wafer bonding or flip chip integration has been more widely used to form III–V photodetectors on silicon, but the bonding approach poses several drawbacks such as the requirements of precise alignment and controlling the bonded interface, and more importantly, the sacrifice of costly III–V wafers which significantly adds up the cost^4,5^.

To overcome such challenges, epitaxy of III–V nanowires on silicon has been proposed as a new approach to monolithically form high-quality III–V on silicon. In nanowire approaches, the small interface area and the unique nanowire geometry allow lateral relaxation of strain, which makes it possible to grow low-defect III–V nanowires on silicon despite the large lattice and thermal mismatches^6,7^. Furthermore, the capability to grow complex structures such as core/shell and axial heterostructures within nanowires wherein the position of nanowires can be controlled by selective-area growth^8^, has enabled realization of novel electronic, optoelectronic, and photonic devices on various substrates including silicon^9–12^. Although III–V nanowires show great promise for heterointegration with silicon, one of the limitations for practical applications is that III–V nanowires typically grow along <111> direction, not < 100> direction. In other words, vertical nanowires can be grown on <111>-oriented wafers, whereas the growth direction of nanowires on <100>-oriented wafers is angled and random^13–15^. Because both the silicon electronics and silicon photonics industry exclusively employ Si (100) or silicon-on-insulator (100) as a standard, the lack of controllability in the growth direction of nanowires on Si (100) substrate undermines the compatibility and degrades the degree of freedom in designing nanowire-based devices on Si (100).

Here, we propose a design of III–V nanowire absorbers on Si (100) platforms which are highly efficient and functional. The proposed design could be fabricated by combining the capability to control the ordering of nanowire arrays on silicon (100) with self-assembled gold plasmonic metasurfaces. Specifically, the orientation of nanowires is aligned to one of the four <111> directions exposed on silicon (100) by employing advanced selective-area epitaxy processes^13,16^ which allows the integration of III–V materials with low defect density despite the lattice mismatch with silicon, and then gold is deposited to form periodic gold nanoholes which are self-assembled to each nanowire. Finite-difference time-domain (FDTD) simulations reveal that high absorption efficiency in III–V nanowires, which greatly exceeds the fill factor of nanowires, can be achieved by exciting plasmonic-photonic hybrid modes. By exploiting both localized surface plasmon (LSP) modes and Bloch-wave surface plasmon polariton (BW-SPP) modes, we demonstrate that the proposed structure can operate not only as infrared photodetectors of high quantum efficiency monolithically integrated on silicon, but as functional sensors that are sensitive to the polarization, wavelengths, and surrounding environment.

## Results

**Figure 1:**
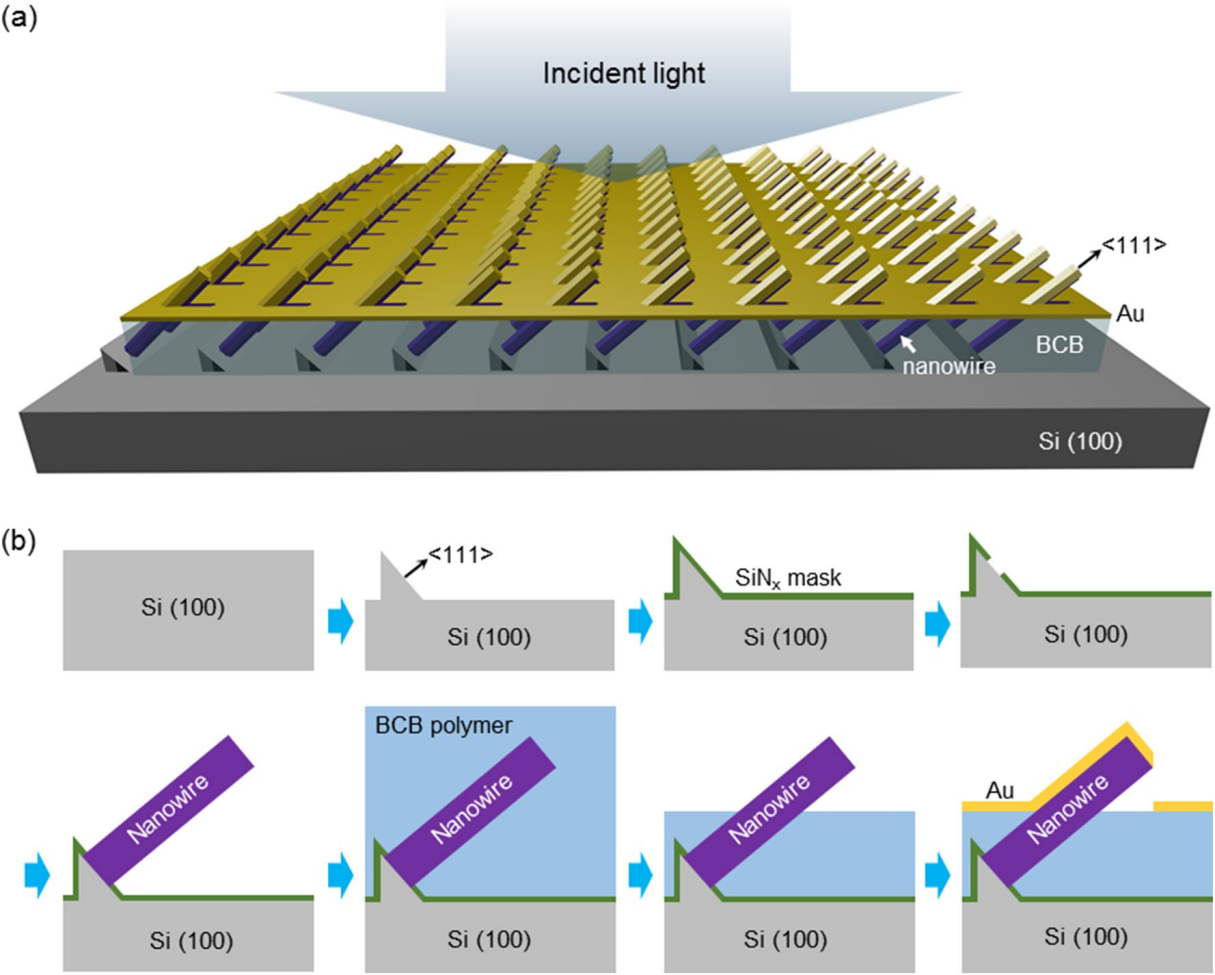
Proposed design. (**a**) Schematic of proposed nanowire-gold hybrid meta-absorber. (**b**) Fabrication processes of the meta-absorber, employing silicon etching, SiN_x_ dielectric masking, III–V nanowire epitaxy, BCB polymer spacer formation, and self-assembly of gold nanostructures by deposition.

The proposed III–V nanowire-gold hybrid meta-absorbers are schematically illustrated in Fig. 1a. On silicon (100) substrate, silicon mesas with <111> sidewalls are formed, and arrays of ordered III–V nanowires are aligned along <111> direction. The nanowires are partially covered by benzocyclobutene (BCB) polymer as a spacer, and gold is deposited on top to form self-assembled nanohole array. Since gold is partially covering the nanowires, where the nanowire facets facing toward the wafer surface are uncovered, this unique gold-nanowire hybrid structure enables the excitation of plasmonic-photonic hybrid modes, which will be further elaborated in the later section.

Although the proposed structure is not experimentally demonstrated here, the fabrication of the structure could be achieved by the following processes. Si (111) planes are first formed on Si (100) wafers by combination of dry and wet etching, as shown in the schematic in Fig. 1b. Then, silicon nitride is deposited as a dielectric mask and patterned to expose silicon for selective-area epitaxy of III–V nanowires. Next, III–V nanowires are epitaxially grown from the exposed area, where the feasibility of forming such well-ordered nanowire arrays on (111) planes of Si (100) is experimentally demonstrated elsewhere recently^13^. Following the growth of nanowire arrays, BCB polymer is used as a dielectric spacer to adjust the height of exposed nanowires and also to electrically isolate the contacts. For this, BCB is first spin-coated and cured, fully covering the nanowires. Due to the viscosity of BCB, the surface of BCB is planar after curing. After BCB is cured, the BCB layer is dry-etched by a standard etch-back process to expose the nanowires, where the exposed height can be precisely controlled^9,17–19^. During the etching of BCB, the directionality and selectivity of etching profiles can be tuned by dry etch conditions to ensure that the BCB underneath the nanowires can be etched away. Lastly, gold is deposited by a standard e-beam evaporation process from vertical direction, which naturally forms self-assembled gold-nanowire hybrid structures with the nanowire half-covered by gold. The other side of the nanowire and the BCB underneath the nanowires not covered by gold, which enables efficient absorption in nanowires by plasmonic resonance.

Compared with previously reported nanowire-based photodetectors and absorbers, the proposed structure has unique advantages in many aspects. Most notably, the nanowires are formed on Si (100) platforms, unlike most of other nanowire-based devices that are formed on <111>-oriented substrates, greatly enhancing the compatibility with standard silicon platforms. Second, the self-assembled gold plasmonic structures can be readily formed by simple evaporation of gold. In previous approaches utilizing vertical nanowires grown on (111) substrates, such gold nanoholes were formed by tilting the sample during the gold deposition^9,17^, wherein the process is more complex and the tilting angle needs to be precisely controlled to meet the design criteria. Third, all nanowires on Si (100) are tilted by 54.7° from the surface normal direction since they are aligned to <111> direction, and the tilting of nanowires further enhances the absorption when combined with vertically deposited gold, which will be discussed in detail in the following section. Therefore, the structure newly proposed here exhibits advanced compatibility and ease of fabrication, with improved efficiency and functionality.

Thus far, the growth of various III–V semiconductor nanowires has been demonstrated on silicon, including GaAs, InGaAs, InAs, GaP and InP nanowires^7,20–24^. Here, we adopt InAs nanowires as the absorber material of interest, since InAs is a small bandgap material that can be used for detection of broad range of infrared wavelengths with high potential for bio- and chemical sensing. Furthermore, the high carrier mobility of InAs nanowires is fascinating for high-speed operation and terahertz applications^25,26^. The proposed hybrid structure is based on semiconducting nanowires with metal and dielectric, and thus both plasmonic resonance at the metal–semiconductor-dielectric junctions and photonic modes within semiconducting nanowires can play roles and excite plasmonic-photonic hybrid modes. Also, the nanowire-gold heterostructures form a periodic array, which represents three-dimensional metasurfaces and can excite BW-SPP modes. Thus, by utilizing these mixed resonance modes, high absorption efficiency and functionalities can be realized, and for this, both the dimensional parameters and the periodicity need to be considered and carefully designed.

**Figure 2:**
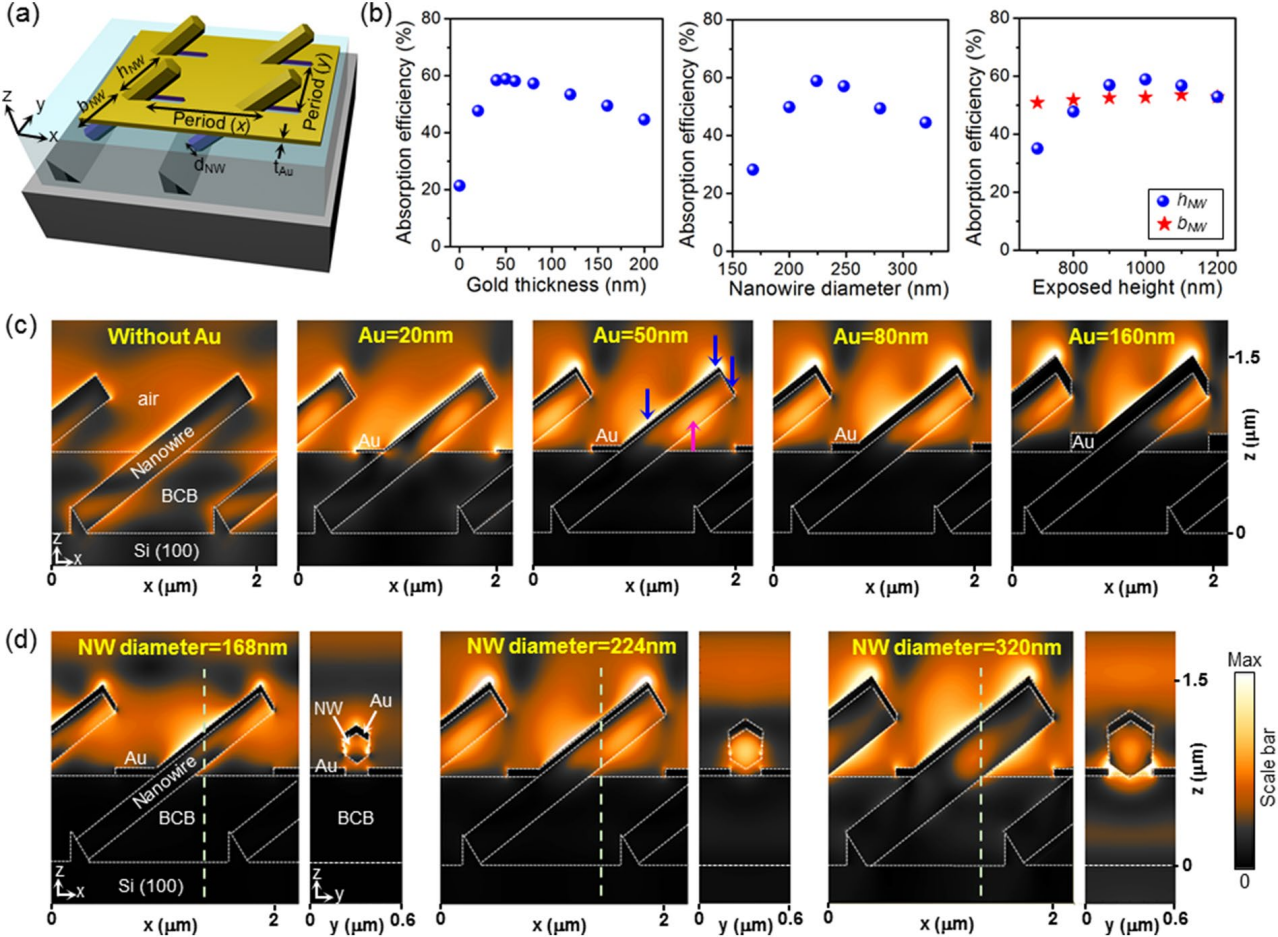
Plasmonic/photonic hybrid mode in nanowires. (**a**) Close-up schematic of meta-absorber structure with dimensional parameters. (**b**) Absorption efficiency of nanowires as a function of gold thickness, nanowire diameter, and exposed/buried nanowire height. (**c**) Electric field profiles (|*E*|) of the structure for various thicknesses of gold, showing the transition from photonic modes to plasmonic modes by increased thickness. (**d**) Electric field profiles (|*E*|) of the structure for various nanowire diameters. The profiles of *yz*-plane are plotted along the dashed lines in *xz*-planes.

We first investigate how the geometry of gold and nanowires affect the excitation of plasmonic-photonic hybrid modes by FDTD simulations. For completeness of the study, major parameters that can be controlled by epitaxy and fabrication, such as the thickness of gold (*t*_*Au*_), diameter of nanowires (*d*_*NW*_), and exposed height (*h*_*NW*_) and buried height (*b*_*NW*_) of nanowires on BCB, are studied as variables, which are shown in Fig. 2a. The detailed simulation setup can be found in the Methods section. Here, it is assumed that the incident light is an *x*-polarized plane-wave source with a wavelength of 1.55 µm from the surface normal direction. The influence of different polarizations and wavelengths are considered in the later section. Unless otherwise noted, the nanowire diameter, exposed height, buried height, and the thickness of gold are set to 224 nm, 1000 nm, 1000 nm, and 60 nm, respectively, with the nanowire periodicity of 1400 nm along the *x*-direction and 680 nm along the *y*-direction. The effect of the gold thickness is first studied, which is one of the most critical factors governing the excitation of plasmonic resonance modes. As shown in Fig. 2b,c, the absorption efficiency of nanowires is low around 20% without gold, since only dielectric modes are excited and the fill factor of nanowires is low. When gold is introduced and the thickness of gold is increased, the absorption efficiency increases to ~ 59% at the thickness range of 40–60 nm, and then monotonically decreases. The electric field intensity profiles in Fig. 2c show the excitation of both a photonic mode inside nanowires (magenta arrow) and plasmonic modes near the gold-nanowire interface (blue arrows). When the gold thickness is further increased, plasmonic modes become dominant, and the absorption in nanowires is decreased due to the absorption loss and reflection by gold. These trends show that the absorption efficiency can be maximized when both plasmonic and photonic modes are excited.

The diameter of nanowires is another key factor governing the absorption. When the diameter of nanowire is too small, then the nanowire cannot support fundamental transverse photonic modes, and only plasmonic modes are excited at the gold-nanowire interface, as shown in the case of the diameter of 168 nm in Fig. 2d. The field profiles plotted in *yz*-plane also confirm this. At the optimal diameter of 224 nm, on the other hand, photonic mode at the center of the nanowire cross-section is notable as well as the plasmonically excited field near the metal-nanowire interface, as shown in the *yz*-plane view in Fig. 2d. At even larger nanowire diameters, the photonic mode becomes more dominant and the total absorption efficiency is decreased. Since maximum absorption in nanowires is achieved at intermediate diameter with confined fields at both the interface and the core of nanowire, this result suggests that exploiting both plasmonic and photonic modes leads to enhanced absorption in nanowires.

Exposed height of nanowires also affects the absorption efficiency, as shown in the graph in Fig. 2b, since both the photonic and plasmonic mode profiles change by the exposed height. On the other hand, the portion of nanowires buried in BCB, *b*_*NW*_, has negligible effect on the total absorption in nanowires, which is shown as red stars in the graph in Fig. 2b. Here, the exposed nanowire height, *h*_*NW*_, is fixed to 1200 nm. The negligible effect of *b*_*NW*_ is because the buried section of nanowires is screened by the gold deposited on BCB surface. Therefore, the absorption properties of the structure can be tuned by adjusting the exposed height, which can be achieved by a BCB etch-back process.

Significant enhancement of nanowire absorption by gold clearly represents that the surface plasmon resonance at the gold-nanowire interface is the key in enhancing the absorption in nanowires. The maximum absorption efficiency is achieved when both the photonic and plasmonic modes are excited, with the maximum absorption efficiency of 59% in nanowires, which is a threefold increase when compared with the case without metal. At this optimized geometry, the absorption by gold is calculated as 25%, which is significantly smaller than the absorption by nanowires. Since the absorption in gold will not contribute to photocurrent when the proposed structure is employed as nanowire photodetectors, such a high absorption in nanowires will be beneficial for infrared photodetectors with high external quantum efficiency by forming Schottky photodetectors or by forming p–n junction within nanowires^25^. We also note that the absorption efficiency of 59% in nanowires is significantly higher than the efficiency that can be achieved from vertical nanowires on <111>-oriented substrates with gold^25^. This is because the nanowires are angled in the proposed design on Si (100) substrates, which is beneficial for enhancing the light-matter interaction from vertically incident light.

**Figure 3:**
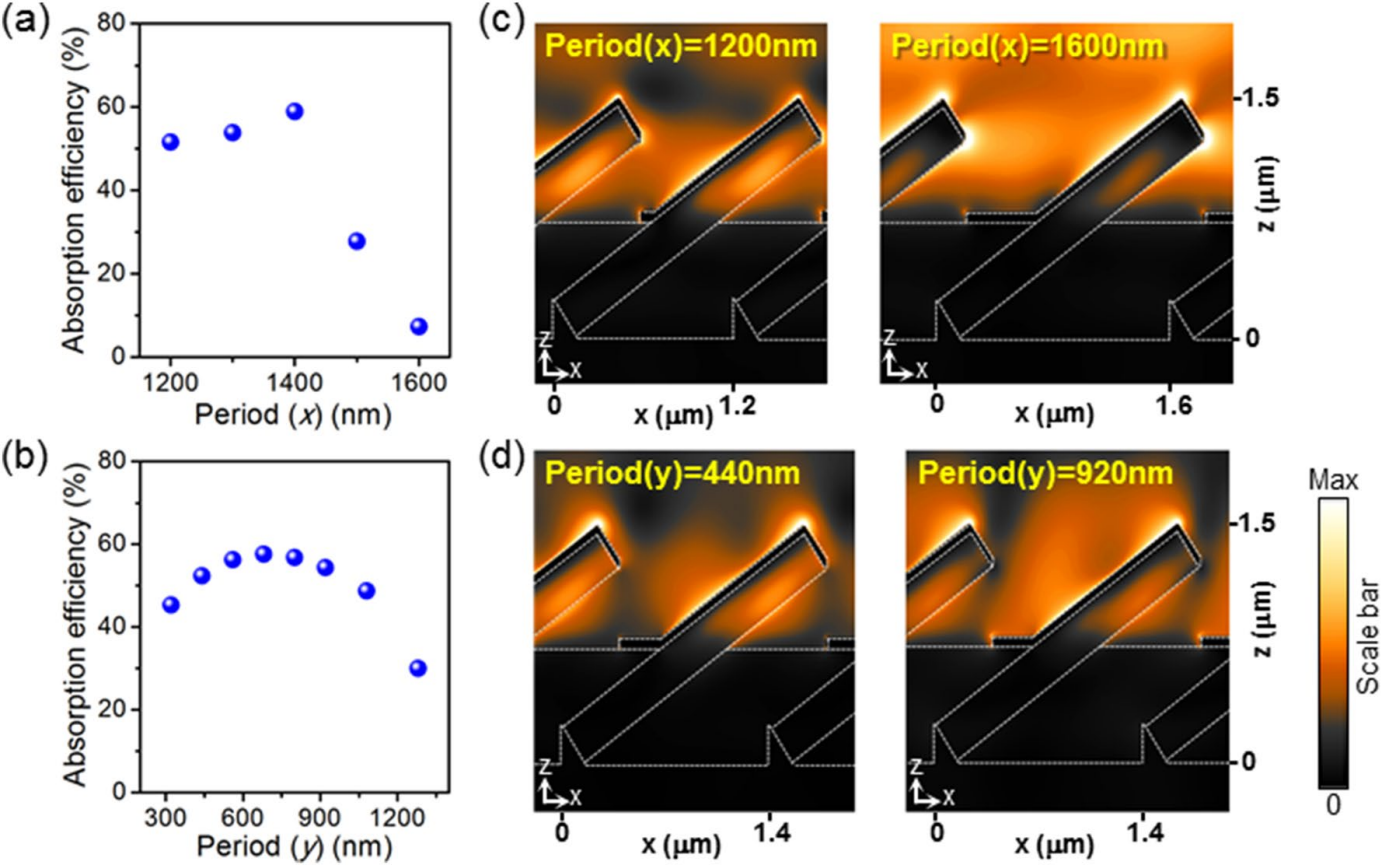
Effect of periodicity of nanowire array. (**a**) Absorption efficiency as a function of periodicity along the *x*-direction and (**b**) *y*-direction. (**c**) Electric field profiles (|*E*|) when periodicity along the *x*-direction and (**d**) *y*-direction is varied.

Next, the effect of periodicity of nanowires on the absorption efficiency is investigated. The periodic arrangement of metal with nanoscale openings can support BW-SPP modes, not only the localized plasmonic resonance considered above, which can further enhance the absorption efficiency by forming plasmonic metasurfaces. Unlike conventional two-dimensional gold nanohole arrays, wherein the resonance wavelength for BW-SPP modes can be simply calculated by the dielectric constants of materials^27,28^, the structure proposed here is three-dimensional, and thus FDTD simulations are employed to find the resonance. When the wavelength of incident light is fixed to 1.55 µm, the maximum absorption efficiency was achieved at the periodicity of 1400 nm and 680 nm for *x*- and *y*- directions, respectively, and the efficiency drops when the periodicity is either increased or decreased (Fig. 3a,b). This unambiguously shows the role of BW-SPP modes in the total absorption; if BW-SPP modes are not involved, the absorption efficiency will only increase by decreasing the periodicity of the array, since the nanowire fill factor (the portion of the area covered by nanowires) will increase by decreased periodicity. In other words, BW-SPP modes excited by the 3D metasurface composed of periodic metal-nanowire heterostructures enhance the absorption of the proposed meta-absorber. The electric field profiles in Fig. 3c,d show that the excited mode profiles are similar regardless of the periodicity and also similar to the case of the periodicity for maximum absorption (diameter of 224 nm in Fig. 2d), except the intensity of fields, showing that the localized plasmonic and photonic modes are not significantly changed by the periodicity of the structures. Considering that the fill factor of the nanowires (*i.e*., the portion of nanowire-covered region from the top view) is only around 16%, the absorption efficiency of 59% achieved here reveals the impact of plasmonic-photonic hybrid modes for enhanced photon absorption.

**Figure 4:**
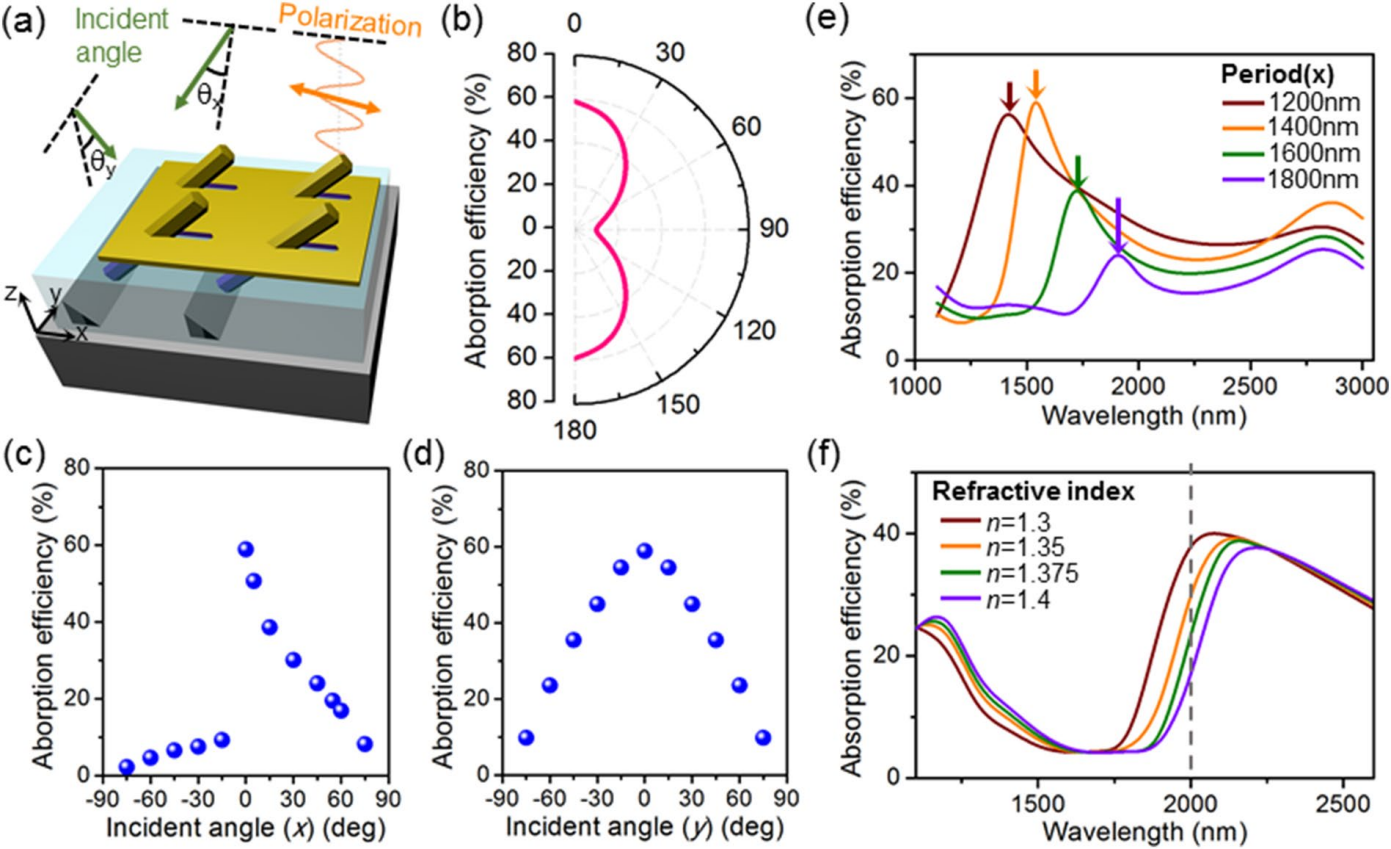
Sensitivity of the meta-absorber. (**a**) Schematic of the polarization and angle of light incident upon the meta-absorber. (**b**) Effect of polarization on the absorption efficiency. 0° represents *x*-polarization. (**c**) Change of absorption efficiency by tilting the incident light along the *x*-axis and (**d**) *y*-axis. (**e**) Broadband absorption spectra of the structure as a function of periodicity, showing the shift of peak wavelength position. (**f**) Broadband absorption spectra of nanowires when the surrounding environment of the structure exhibits refractive index other than unity.

With the proposed metasurface absorber structures, we show the functionality and feasibility for their implementation in various applications that are difficult to realize by conventional photodetectors. In conventional thin film-based photodetectors which are most widely used, the detector is generally sensitive only to the light intensity, but insensitive to other features such as polarization, incident angle, small change of photon wavelengths, or surrounding environment^29^. On the contrary, the proposed absorber on silicon utilizes surface plasmon resonances and BW-SPP resonances of periodic 3D structures, and thus the situation is vastly different, making the proposed device more suitable for diverse applications requiring sensitivities to those parameters. For example, when the polarization or incident angle of the light source is changed, as shown in Fig. 4a, the absorption in the nanowires is abruptly changed as well. The absorption efficiency is maximum around 59% when the light is *x*-polarized, while the efficiency drops to 8.9% under the same intensity of light when the beam is *y*-polarized (90° in Fig. 4b). Also, tilting of the incident beam angle from the surface normal direction changes the wavevector and greatly modulates the excitation of plasmonic resonance at the gold-nanowire interface. This is manifested as the change of absorption efficiency (Fig. 4c,d) and is more drastic when tilted along the *x*-direction.

Furthermore, because the proposed metasurface absorber structure employs BW-SPP resonances from periodic arrays, the peak absorption wavelength can be tuned by changing the periodicity of the array. In conventional thin film-based III–V photodetectors, tunability can be also achieved using same principles by nanopatterning gold on the surface^30^. However, monolithic integration on silicon platforms is challenging due to high lattice mismatch (*e.g*. mismatch of 11% between InAs and Si), and thus the proposed approach of employing nanowires, which can be grown with high material quality on silicon, with self-assembled gold plasmonic arrays provides a new path toward direct integration of functional and efficient infrared photodetectors on silicon platforms. The absorption efficiency as a function of wavelengths in Fig. 4e shows that the peak absorption wavelengths (arrows in Fig. 4e) shift following the periodicity of the array. When the periodicity of the nanowires along the *x*-direction is increased from 1200 to 1800 nm, the peak absorption wavelength also monotonically increases from ~ 1400 to ~ 1900 nm. This means that multi-spectral detectors could be formed with a single element of InAs nanowires on a single silicon chip, by making focal plane arrays with varying periodicities of InAs nanowires, so that each pixel composed of nanowire arrays with different periodicities shows photoresponse sensitive to corresponding wavelengths.

Lastly, we show the sensitivity of proposed platform to surrounding environment, which makes the platform promising for sensing applications. Thus far, it is assumed in FDTD simulations that the meta-absorber structure is in air. On the other hand, when the nanowires are surrounded by materials with the refractive index other than unity, both the localized and Bloch-wave mode profiles are altered significantly. As a result, the wavelength-dependent absorption efficiencies in Fig. 4f show that the absorption spectra change significantly when the refractive index of surrounding environment is changed from unity (*i.e*., air or vacuum) to 1.3–1.4. Also, within this range, which is chosen as an example, the absorption peak shifts significantly by a small change of the index, which leads to a decrease of the absorption efficiency from > 40 to 15% when the refractive index is changed from 1.3 to 1.4 at the light wavelength of 2 µm (dashed line in Fig. 4f). Thus, we envision that the proposed meta-absorber could potentially be used for environmental or biochemical monitoring by detecting the change of refractive index. Although conventional metasurfaces based on metal-dielectric structures exhibit much higher wavelength sensitivity and better tunability in terms of absorption, transmission or reflection^31,32^, the proposed plasmonic structure based on metal-semiconducting nanowire could be more suitable for functional on-chip photodetectors because most of the absorption takes place in nanowires which can contribute to photocurrent, unlike the case of metal-dielectric structures where the metal is the absorber. We also envision that the proposed design can be developed as a basic element in in-vivo imaging system (IVIS), wherein the imaging depth and resolution could be greatly enhanced by selecting the infrared wavelength of interest depending on the type of imaging element, with optimized geometry of nanowire arrays^33,34^. Lastly, by functionalizing gold, the sensitivity to specific targets could be further increased in bio- and chemical sensing platforms^35^. Therefore, the proposed nanowire-based meta-absorber design on silicon chip provides possibilities for various applications due to its high functionality, design flexibility, and sensitivity.

## Discussion

In summary, we have proposed a III–V nanowire array-based meta-absorber formed by selective-area epitaxy of nanowires on silicon (100) platform and self-assembly of gold on nanowires. The proposed plasmonic-photonic hybrid structure exhibits high absorption efficiency by utilizing both localized and periodic plasmonic modes at the metal-nanowire interface, as well as photonic modes in nanowires. Furthermore, the proposed absorber exhibits high sensitivity to the geometry, incident light, and surrounding environment. Therefore, the proposed III–V nanowire-based absorbers on silicon (100) could be utilized in diverse imaging and sensing applications with high efficiency and sensitivity.

## Methods

FDTD simulations are conducted using a software, Lumerical FDTD Solutions. The material properties of Si, InAs and gold in simulations are adopted from^36–38^, and the refractive index of BCB is set to 1.535. The size of a unit cell is set by the periodicity of the nanowire array. Bloch boundary conditions are used for the *x*- and *y*-directions, and perfectly matched layer (PML) condition is used for the z-direction. The size of simulation meshes is tuned to be small enough for accurate results, which is confirmed by verifying the convergence of simulation results by varying the size of meshes. The absorption efficiency of nanowires is calculated by dividing the absorbed energy in nanowires by that of the plane-wave source.
